# Secondary cardiocerebral infarction after acute inferior myocardial infarction treated with two emergency thrombectomies: a case report

**DOI:** 10.3389/fmed.2025.1690729

**Published:** 2025-11-20

**Authors:** Ming-Zhen Dong, En-Bo Zhu, Ming-Quan Lin, Guang-Hui Dong, Guang-Lin Jin, Lin-Zhuo Qu, Hui-Ying Che, Hong-Jian Guan

**Affiliations:** 1 Department of Neurology, Yanbian University Hospital, Yanji, Jilin, China; 2 Department of General Medicine, Yanbian University Hospital, Yanji, Jilin, China

**Keywords:** acute ischemic stroke, cardiocerebral infarction, acute inferior myocardial infarction, emergency thrombectomies, rare case report

## Abstract

Acute myocardial infarction (AMI) and acute ischemic stroke (AIS) are among the leading causes of disability and mortality worldwide. Cases of acute ischemic stroke secondary to acute inferior myocardial infarction are exceedingly rare in clinical practice, leaving physicians with limited experience and lacking established guidelines for management. This report presents the case of an elderly female patient who was admitted with acute inferior myocardial infarction. During conservative management, she subsequently developed two episodes of acute ischemic stroke, both of which were treated with emergency endovascular thrombectomy, and she ultimately recovered and was discharged.

## Introduction

Cardiovascular and cerebrovascular diseases remain the leading causes of morbidity and mortality worldwide (1). When acute inferior myocardial infarction and acute ischemic stroke occur simultaneously, the patient’s overall prognosis deteriorates significantly, and the mortality rate increases markedly (2, 3). The simultaneous occurrence of acute ischemic stroke and acute myocardial infarction within 48 h is defined as cardiocerebral infarction (CCI), a condition associated with a high risk of disability and up to an eightfold increase in mortality (4, 5). The concept of CCI was first proposed by Omar et al. in 2010. Despite this, cases of acute ischemic stroke following acute inferior myocardial infarction remain exceedingly rare in clinical practice (6). Due to the scarcity of such cases, clinicians often face major challenges in selecting optimal treatment strategies—particularly in determining whether emergency thrombectomy is required—thus placing considerable challenges to clinical decision-making.

Here, we report a rare case of an elderly female patient who developed two episodes of acute ischemic stroke secondary to acute inferior myocardial infarction, both successfully treated with emergency thrombectomy. This case aims to provide insights and potential guidance for future clinical practice.

## Case presentation

**Figure 1:**
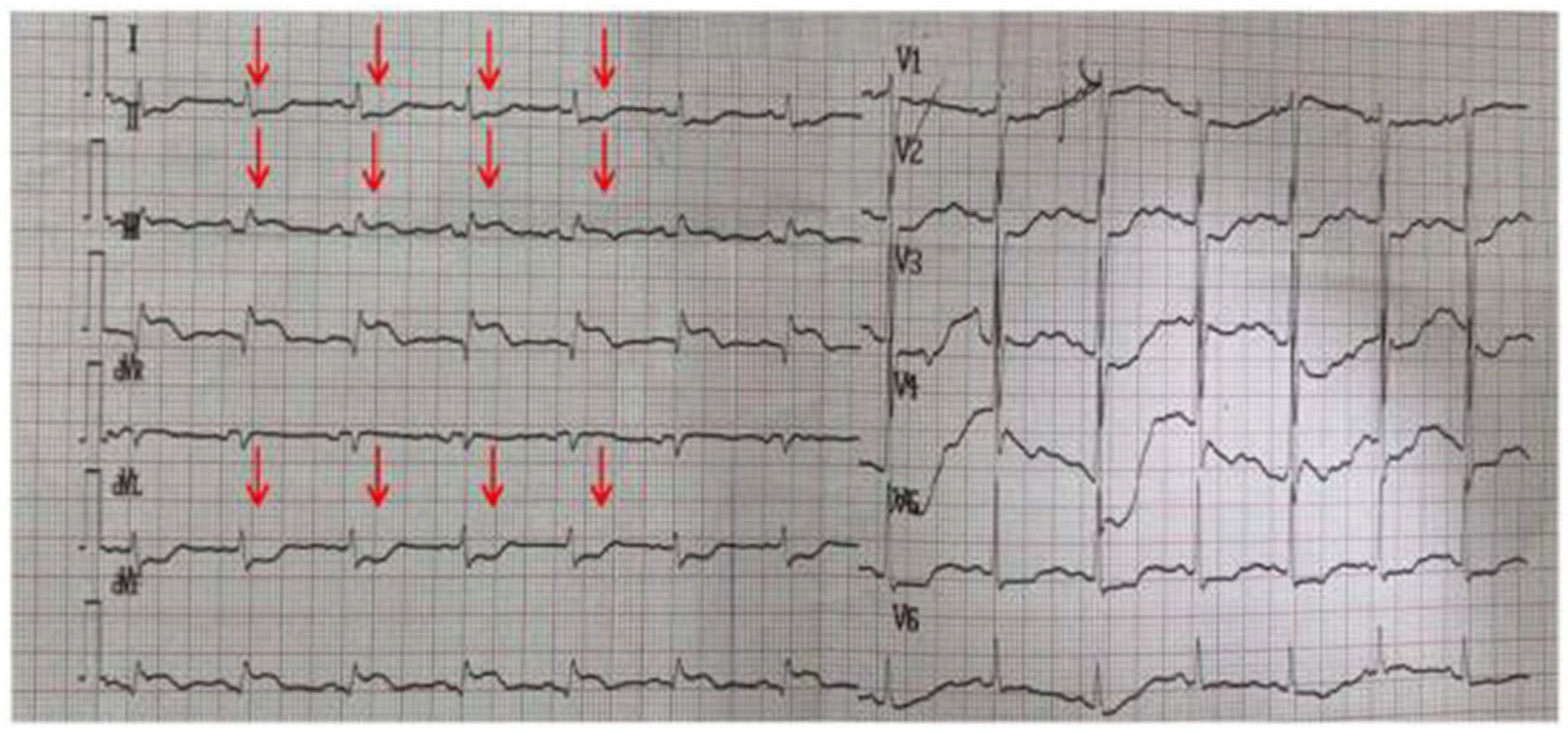
Electrocardiogram showing sinus rhythm with ST-segment elevation and T-wave inversion in leads II, III, and aVF.

**Figure 2:**
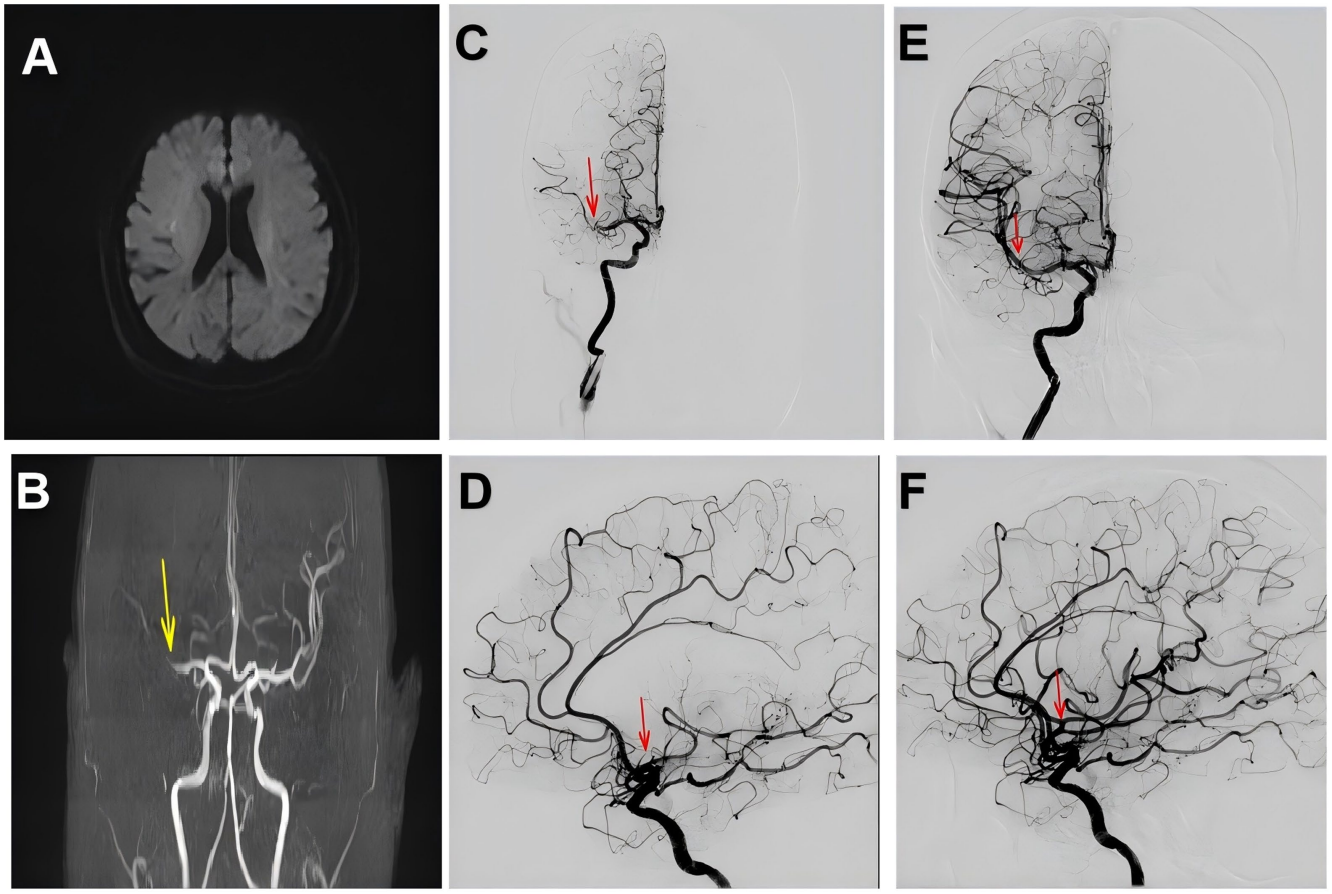
**(A)** Brain MRI showing multiple punctate abnormal signal foci in the periventricular and centrum semiovale regions bilaterally. **(B)** Brain MRA demonstrating stenosis of the right middle cerebral artery (M1 segment) indicated by the yellow arrow, with reduced distal branches. **(C,D)** Right cerebral angiography showing occlusion of the right middle cerebral artery. **(E,F)** Right cerebral angiography showing restoration of blood flow in the right middle cerebral artery, indicated by the red arrow indicated by the red arrow.

A 75-year-old woman was admitted to the hospital with a chief complaint of intermittent chest tightness and chest pain for 3 years, which had worsened and was accompanied by shortness of breath for the past 5 days. After the aggravation of chest pain, she experienced sweating, dizziness, and back pain. Each episode of pain lasted about 1 h and was more severe at night. On admission, her heart rate was 72 beats per minute, and blood pressure (BP) was 135/68 mmHg. Electrocardiography (ECG) showed sinus rhythm with ST-segment elevation and T-wave inversion in leads II, III, and aVF (Figure 1). Myocardial injury markers were as follows: CK-MB 3.99 ng/mL, cTnI 8.64 ng/mL, and myoglobin (Myo) 18.42 ng/mL. NT-proBNP was 2800.96 ng/L. Echocardiography revealed a left ventricular ejection fraction (LVEF) of 54%, with segmental wall motion abnormalities—specifically, reduced wall motion amplitude and thickening rate in the basal to mid segments of the left ventricular posterior wall, while the right ventricular wall thickness remained normal and the three-layer myocardial structure was preserved. No obvious abnormalities were found in the remaining wall segments. Additionally, moderate mitral regurgitation, borderline pulmonary hypertension, and a trace pericardial effusion were observed. The patient had a medical history of hypertension, type 2 diabetes mellitus, and cerebral infarction. After treatment for the prior stroke, no residual neurological deficits remained. The final diagnoses were as follows: (1) Acute coronary syndrome — acute ST-segment elevation myocardial infarction (inferior wall), Killip class II; (2) Stage 1 Hypertension; (3) Type 2 diabetes mellitus. Because the patient’s symptoms had persisted for more than 24 h before admission, percutaneous coronary intervention (PCI) was not performed. After 18 h of medical therapy in the cardiology department, the patient suddenly developed mouth deviation and weakness of the left limbs. MRI and MRA revealed multiple patchy abnormal signals in the periventricular and centrum semiovale regions, with right middle cerebral artery (MCA) M1 segment stenosis and reduced distal branches (Figures 2A,B). Neurological examination revealed somnolence, dysarthria, shallow left nasolabial fold, right-sided mouth deviation, tongue deviation to the left, left upper limb muscle strength of grade 1, and left lower limb muscle strength of grade 4 (NIHSS score = 9). As the onset time was within the therapeutic window, emergency endovascular thrombectomy was performed after obtaining informed consent. Digital subtraction angiography (DSA) revealed right MCA occlusion (Figures 2C,D). A microcatheter was advanced over a microwire into the M2 segment of the right MCA, and a 4.0 × 30 mm stent retriever was deployed for 5 min. With simultaneous aspiration using a Stryker intracranial support catheter, a large thrombus was retrieved, and repeat angiography confirmed recanalization of the right MCA with mTICI grade ≥2B (Figures 2E,F). The procedure was terminated, and the patient was transferred to the NICU. After treatment, her consciousness improved, and left-sided motor strength recovered to grade 4 before she was transferred to the general ward.

**Figure 3:**
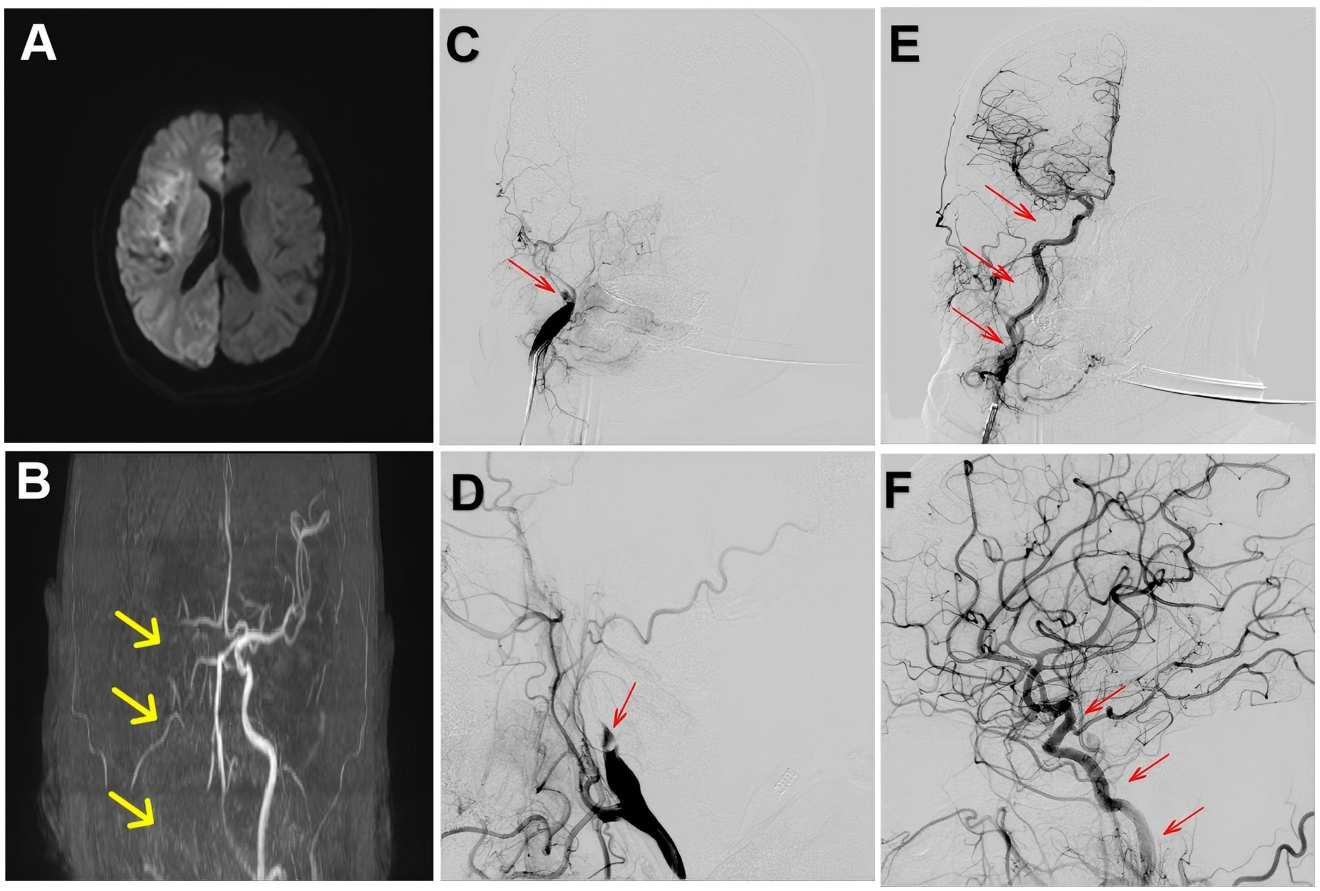
**(A)** Brain MRI showing a large infarction in the right cerebral hemisphere. **(B)** Brain MRA demonstrating occlusion of the right internal carotid artery, indicated by the yellow arrow. **(C,D)** Right cerebral angiography showing occlusion of the right internal carotid artery, indicated by the red arrow. **(E,F)** Right cerebral angiography showing restoration of blood flow in the right internal carotid artery, indicated by the red arrow.

Four days later, the patient experienced sudden unconsciousness. Examination revealed somnolence, unresponsiveness, left pupil 2.0 mm with absent light reflex, right pupil unassessable, shallow left nasolabial fold, right-sided mouth deviation, tongue immobility, and inability to assess limb strength (NIHSS score = 29). Emergency MRI and MRA showed a large infarction in the right cerebral hemisphere with occlusion of the right internal carotid artery (ICA) (Figures 3A,B). A second emergency thrombectomy was performed after family consent. DSA confirmed right ICA occlusion (Figures 3C,D). After balloon occlusion of antegrade flow, a 6.0 × 30 mm stent retriever was deployed distal to the occlusion. Repeat angiography showed thrombus in the cavernous segment of the right ICA, and the stent was maintained for 5 min. With aspiration using the Stryker catheter, a large thrombus was extracted, and repeat angiography demonstrated successful recanalization of the right ICA with mTICI grade ≥2B (Figures 3E,F). During the patient’s return to the NICU for treatment, we performed tests for anti-neutrophil cytoplasmic antibodies (ANCA), antinuclear antibodies (ANA), rheumatoid factor (RF), and coagulation times, all of which showed no significant abnormalities. The deep vein thrombosis (DVT) assessment indicated a high risk, and We implemented appropriate measures to prevent venous thrombosis. Following 10 days of treatment, neurological examination revealed dysarthria, shallow right nasolabial fold, buccal leakage on puffing, tongue deviation to the right, and motor strength recovery to grade 4 (NIHSS score = 9). The patient continued treatment in the NICU and was later transferred back to the cardiology department. After an additional 7 days of therapy, she was discharged with residual right-sided facial palsy and mild motor impairment (NIHSS score = 5).

The patient was advised to continue oral medications after discharge, including enteric-coated aspirin, clopidogrel sulfate tablets, atorvastatin calcium tablets, metoprolol succinate sustained-release tablets, isosorbide mononitrate tablets, spironolactone tablets, and furosemide tablets, and to attend regular follow-up visits. During follow-up, the patient’s 90-day modified Rankin Scale (mRS) score was 3, indicating a relatively favorable outcome for this case.

## Discussion

The simultaneous occurrence of AMI and AIS is extremely rare (7). Yeo et al. (8) reported that the incidence of concurrent acute inferior myocardial infarction and AIS is only 0.0009%. Although evidence indicates that AMI is a risk factor for AIS, with the first 24 h after onset being the period of highest vulnerability, the 1-year risk of developing AIS after AMI is estimated at 4. 1% (9, 10). Furthermore, patients with Killip class II have a markedly increased risk of AIS. However, in this case, the patient experienced two episodes of AIS during the course of an acute inferior myocardial infarction, an exceptionally uncommon form of CCI (11). The main mechanisms underlying CCI include cardiac embolism, atherosclerosis, neurogenic factors, inflammatory responses, and certain systemic diseases (12). In clinical practice, central brain infarctions are often associated with cardioembolic events, particularly in patients with atrial fibrillation; however, this patient had no history of atrial fibrillation. Echocardiography revealed reduced motion amplitude and decreased thickening rate in the basal to mid segments of the left ventricular posterior wall. Based on these findings, we strongly suspect that the embolus may have originated from a left ventricular thrombus formed due to segmental wall motion abnormalities or hypokinesis, leading to acute ischemic stroke. Similar cases have also been reported in previous studies (13, 14). Although left ventricular thrombus is a known complication of AMI, it is still relatively uncommon, typically occurring in dilated cardiomyopathy and acute anterior wall myocardial infarction. This case demonstrates that acute inferior myocardial infarction, though rare, can also result in large-vessel occlusion AIS (15, 16).

Previous studies have shown that the treatment rate of asynchronous CCI, such as in the present case, is significantly lower than that of synchronous CCI (17). In 2018, the American Heart Association/American Stroke Association (AHA/ASA) recommended that in simultaneous CCI, alteplase may be administered at the same dose as for cerebral ischemia, followed by percutaneous coronary intervention (18). However, the patient described here developed AIS 18 h after the onset of acute inferior myocardial infarction. For such cases, no established guidelines or standardized treatment strategies are currently available.

This case helps to fill an important gap in clinical understanding. Although echocardiography did not reveal a thrombus in the left ventricle, the examination was performed after the onset of cerebral infarction, and the thrombus may have already dislodged, resulting in the absence of a detectable thrombus image. However, the echocardiogram showed extensive hypokinesis of the left ventricular wall. Other tests and clinical findings ruled out alternative sources of emboli, leading us to strongly suspect that acute inferior myocardial infarction caused regional left ventricular wall motion abnormalities, resulting in thrombus formation, which subsequently embolized and triggered an acute ischemic stroke. The patient’s second acute ischemic stroke was similarly caused by left ventricular thrombus formation due to persistent wall motion abnormalities, leading to occlusion of the internal carotid artery after embolization. For this rare case, emergency mechanical thrombectomy achieved a favorable outcome, providing clinicians with a clear and effective therapeutic approach for managing cardio-cerebral infarction, particularly the rarer scenario of acute ischemic stroke following acute inferior myocardial infarction.

## Conclusion

Although CCI is extremely rare and most often results from acute anterior myocardial infarction complicated by atrial fibrillation leading to acute ischemic stroke, this case demonstrates that acute inferior myocardial infarction can also give rise to ventricular thrombus due to wall motion abnormalities, ultimately resulting in AIS. Moreover, patients with left ventricular thrombus may experience recurrent large-vessel occlusions. In such situations, clinicians may take this case as a reference and actively consider emergency endovascular thrombectomy to improve patient survival and prognosis.

## Data Availability

The original contributions presented in the study are included in the article/Supplementary material, further inquiries can be directed to the corresponding authors.

## References

[1] Virani SS Alonso A Benjamin EJ Virani SS Benjamin EJ Bittencourt MS . Heart disease and stroke statistics-2020 update: a report from the American Heart Association. Circulation. (2020) 141:e139–596. doi: 10.1161/CIR.0000000000000757, PMID: 31992061

[2] Lodeen H Esmati S Okan T Arastu A Vilendecic D Singh G . The simultaneous occurrence of acute ST-elevation myocardial infarction, acute ischemic stroke, and pulmonary embolism. Cureus. (2023) 15:e44222. doi: 10.7759/cureus.44222, PMID: 37767245 PMC10522405

[3] Abdi IA Karataş M Abdi AE Hassan MS Yusuf Mohamud MF. Simultaneous acute cardio-cerebral infarction associated with isolated left ventricle non-compaction cardiomyopathy. Ann Med Surg (Lond). (2022) 80:104172. doi: 10.1016/j.amsu.2022.104172, PMID: 36045823 PMC9422203

[4] Ibekwe E Kamdar HA Strohm T. Cardio-cerebral infarction in left MCA strokes: a case series and literature review. Neurol Sci. (2022) 43:2413–22. doi: 10.1007/s10072-021-05628-x, PMID: 34590206 PMC8480750

[5] Mehta S Kakouros N Mir T Loree S Qureshi W. Prevalence and outcomes of patients with acute ischemic stroke with concomitant ST-segment-elevation myocardial infarction (results from national inpatient sample 2016-2019). Stroke. (2024) 55:1245–53. doi: 10.1161/STROKEAHA.123.044550, PMID: 38529635

[6] Bao CH Zhang C Wang XM Pan YB. Concurrent acute myocardial infarction and acute ischemic stroke: case reports and literature review. Front Cardiovasc Med. (2022) 9:1012345. Published 2022 Nov 1. doi: 10.3389/fcvm.2022.1012345, PMID: 36386323 PMC9663457

[7] Aggarwal G Patlolla SH Aggarwal S Cheungpasitporn W Doshi R Sundaragiri PR . Temporal trends, predictors, and outcomes of acute ischemic stroke in acute myocardial infarction in the United States. J Am Heart Assoc. (2021) 10:e017693. doi: 10.1161/JAHA.120.017693, PMID: 33399018 PMC7955313

[8] Omar HR Fathy A Rashad R Helal E. Concomitant acute right ventricular infarction and ischemic cerebrovascular stroke; possible explanations. Int Arch Med. (2010) 3:25. doi: 10.1186/1755-7682-3-25, PMID: 20977759 PMC2974668

[9] Ng TP Wong C Leong ELE Tan BY Chan MY Yeo LL . Simultaneous cardio-cerebral infarction: a meta-analysis. QJM. (2022) 115:374–80. doi: 10.1093/qjmed/hcab158, PMID: 34051098

[10] Murakami T Sakakura K Jinnouchi H Taniguchi Y Tsukui T Watanabe Y . Acute ischemic stroke and transient ischemic attack in ST-segment elevation myocardial infarction patients who underwent primary percutaneous coronary intervention. J Clin Med. (2023) 12:840. doi: 10.3390/jcm12030840, PMID: 36769488 PMC9917385

[11] Hurskainen M Tynkkynen J Eskola M Lehtimäki T Hernesniemi J. Risk factors for ischemic stroke after acute coronary syndrome. J Am Heart Assoc. (2023) 12:e028787. doi: 10.1161/JAHA.122.028787, PMID: 37421266 PMC10382101

[12] Gao W Yu L She J Sun J Jin S Fang J . Cardio-cerebral infarction: a narrative review of pathophysiology, treatment challenges, and prognostic implications. Front Cardiovasc Med. (2025) 12:1507665. Published 2025 Mar 25. doi: 10.3389/fcvm.2025.1507665, PMID: 40201791 PMC11975930

[13] Watanabe T Kobara S Amisaki R Yamamoto K. Primary percutaneous coronary intervention for cardio-cerebral infarction: a case report. Front Cardiovasc Med. (2023) 10:1165735. Published 2023 Jul 31. doi: 10.3389/fcvm.2023.1165735, PMID: 37583581 PMC10424438

[14] Hattori Y Ikeda S Matsumoto M Tagawa N Hatakeyama K Ihara M. Case report: postmortem brain and heart pathology unveiling the pathogenesis of coexisting acute ischemic stroke and electrocardiographic abnormality. Front Cardiovasc Med. (2023) 10:1200640. doi: 10.3389/fcvm.2023.1200640, PMID: 37388637 PMC10306394

[15] Cheong XK Tan JK Kang Z Kori N Periyasamy PR. A difficult case of cardio-cerebral infarction syndrome with left ventricular thrombus. Cureus. (2024) 16:e60196. doi: 10.7759/cureus.60196, PMID: 38868266 PMC11168241

[16] Terasawa Y Miyamoto Y Kohriyama T. Features of cerebral infarction due to left ventricular Thrombus. Intern Med. (2022) 61:2581–5. doi: 10.2169/internalmedicine.8015-21, PMID: 36047094 PMC9492482

[17] Ho JS Zheng H Tan BY Ho AF Foo D Foo LL . Incidence and outcomes of Cardiocerebral infarction: a cohort study of 2 National Population-Based Registries. Stroke. (2024) 55:2221–30. doi: 10.1161/STROKEAHA.123.044530, PMID: 39082144

[18] Eskandarani R Sahli S Sawan S Alsaeed A. Simultaneous cardio-cerebral infarction in the coronavirus disease pandemic era: a case series. Medicine (Baltimore). (2021) 100:e24496. doi: 10.1097/MD.0000000000024496, PMID: 33530272 PMC7850703

