# Supplementary material for: Secondary cardiocerebral infarction after acute inferior myocardial infarction treated with two emergency thrombectomies: a case report
Source: Front Med (Lausanne). 2025 Nov 20;12:1690729. doi: 10.3389/fmed.2025.1690729 (PMC12679706; doi:10.3389/fmed.2025.1690729)
Supplement: Supplementary file 1 [file Data_Sheet_1.PDF]

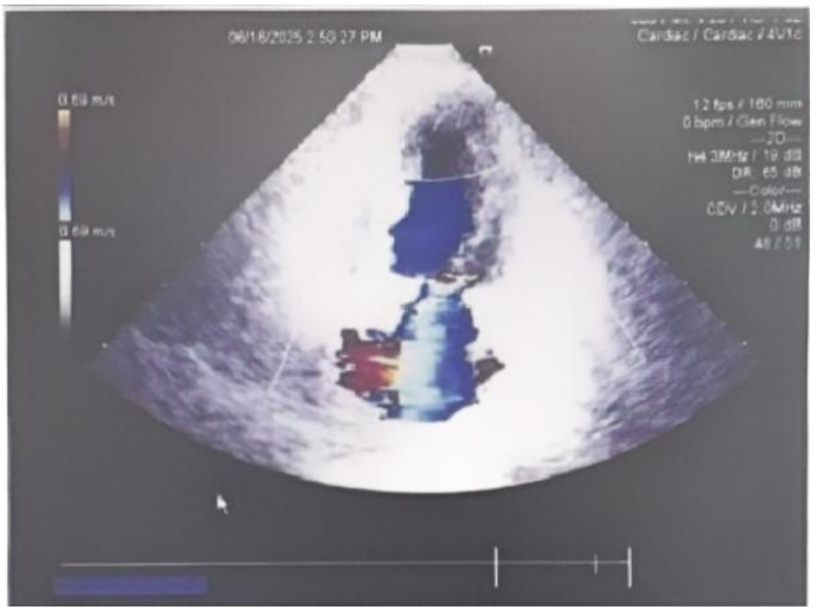

超声所见：

超声测值：

升主动脉内径32 mm。主动脉根部内径30 mm。 左房内径38 mm。左室舒末内径47 mm。 左室收末内径33 mm。 室间隔厚度9mm。左室后壁厚径9 mm。主肺动脉内径22mm。

心功能：EF54%。FS27 %。

Doppler: 肺动脉瓣0. 7 m/s。主动脉瓣1. 0 m/s。 二尖瓣口：E峰0. 8 m/s。A峰0. 9m/s。DT204 ms, 三尖瓣返流速度3. 0 m/s。右室压力40 mmHg。

组织多普勒 (DTI): 二尖瓣环与左室侧壁交界处频谱示： e’:6cm/s、a';8cm/s,E/e'<14

2D:各房室腔内径正常范围。室间隔及左、右室壁厚度正常，左室后壁基底段至中间段，运动幅度减低，室壁增厚率减低，室壁三层结构存在，余室壁节段运动未见明确异常。房、室间隔连续完整。各瓣膜形态、结构、启闭运动未见明显异常。大动脉关系、内径正常。心尖部心包腔内舒张期可探及无回声暗区2mm.

CDFI:二尖瓣中量反流束，三尖瓣、主动脉瓣、肺动脉瓣见微量反流束。

超声提示：

节段性室壁运动异常

二尖瓣中量反流

肺动脉高压临界值

心包积液（微量）

To facilitate the editor's review of the patient's echocardiographic findings, I have translated the echocardiography report into English for your reference.

Echocardiographic Findings

Measurements:

Ascending aorta diameter: 32 mm;Aortic root diameter: 30 mm;Left atrial diameter: 38 mm;Left ventricular end-diastolic diameter: 47 mm;Left ventricular end-systolic diameter: 33 mm;Interventricular septal thickness: 9 mm;Left ventricular posterior wall thickness: 9 mm;Main pulmonary artery diameter: 22 mm.

Cardiac function:

Ejection Fraction (EF): 54%;Fractional Shortening (FS): 27%.

Doppler:

Pulmonary artery velocity: 0.7 m/s;Aortic velocity: 1.0 m/s;Mitral inflow velocity: E = 0.8 m/s, A = 0.9 m/s;Deceleration time (DT): 204 ms;Mitral regurgitation velocity: 3.0 m/s;Right ventricular systolic pressure (RVSP): 40 mmHg.

Tissue Doppler Imaging (DTI):

Mitral annulus at the lateral wall: e' = 6 cm/s, a' = 8 cm/s, E/e' < 14.

2D Findings:

The size and shape of the left and right atria and ventricles are within normal limits. The interventricular septum and left ventricular posterior wall are normal in thickness. The motion of the basal to mid-inferior wall of the left ventricle is reduced, with hypokinesis noted. The other left ventricular wall segments show normal motion and morphology. The structure and motion of the aortic valve, mitral valve, and tricuspid valve are normal, with no prolapse observed. The pericardium appears normal. The aortic root and ascending aorta are normal in morphology and diameter. During diastole, a small echo-free space (~2 mm) is seen in the pericardial area near the apex.

Color Doppler Flow Imaging (CDFI):

Mild mitral regurgitation;Mild tricuspid regurgitation;Mild pulmonary regurgitation.

Echocardiographic Impression

Abnormal left ventricular wall motion in the inferior wall
Mild mitral regurgitation
Mild pulmonary hypertension
Mild pericardial effusion (posterior)

序号	代号	项目名称	结果	单位	参考范围
1	MPO	过氧化物酶抗体	<20	RU/ml	<20
2	PR3	抗蛋白酶3抗体	<20	RU/ml	<20
3	GBM	抗肾小球基底膜抗体	<20	RU/ml	<20
4	pANCA	抗中性粒细胞胞浆抗体：核周型	阴性	—	<1:10
5	cANCA	抗中性粒细胞胞浆抗体：胞浆型	阴性	—	(1:10
6	aANCA	抗中性粒细胞胞浆抗体；不典型	阴性	—	(1:10

序号	代号	项目名称	结果	单位	参考范围
	nRNP/Sm	抗核糖核蛋白nRNP/Sm抗体	<2.00	RU/ml	0-20
2	Sa	抗Sm抗体	<2.00	RU/ml	0-20
3	SSA	抗SSA抗体	<2.00	RU/ml	0-20
4	SSB	抗SSB扰体	〈2.00	RU/ml	0-20
5	Ro-52	抗Ro52抗体	〈2.00	RU/ml	0-20
6	Scl-70	抗ScJ-70抗体	<2.00	RU/ml	0-20
7	Jo-1	抗组胺酰LRNA合成酶抗体	<2.00	RU/ml	0-20
8	ANA	抗核抗体(核型)	阴性		阴性
9	ANA	抗核抗体(滴度)	阴性		《1:100
10	dsDVA	抗双链脱氧核糖核酸抗体	阴性		<1:10

序号	代号	项目名称	结果	单位	参考范围
1	RF	类风湿因子	7.70	IU/mL	0-15
2	IgG	HR免疫球蛋白G	9.35	g/1	7-16
3	IgA	HR免疫球蛋白A	5.55	f g/L	0.7—4
4	IgM	HR免疫球蛋白M	0.62	g/L	0.4—2.3
5	C3	HR补体C3	1.21	g/L	0.9—1,8
6	C4	HR补体CA	0.25	g/L	0.1—0.4

序号	代号项目名称	结果	单位	参考范围
1	PT-T 凝血酶原时间(PT)	13.30	Sec	10-15
2	PT-%凝血酶原时间活度(PT%)	102.0	%	70-130
3	PT-1NR 国际标准化比值(PTINR)	0.99		0.71—1.3
4	APTT-T 部分凝血馥原时间(APTT)	31.4	Sec	20--43.5
5	FBGC纤维蛋白原定量(FBGC)	4.29	g/L	2-4
6	TT 凝血酶时间(TT)	21.5	1 Sec	14-20
